# Immunological aspects of the efficiency of protectotype vaccination strategy against chicken infectious bronchitis

**DOI:** 10.1186/s12917-017-0963-1

**Published:** 2017-02-08

**Authors:** Marcin Smialek, Bartlomiej Tykalowski, Daria Dziewulska, Tomasz Stenzel, Andrzej Koncicki

**Affiliations:** Departemnt of Poultry Diseases, Faculty of Veterinary Medicine, University of Warmia and Mazury, Oczapowskiego 13, Olsztyn, 10-719 Poland

**Keywords:** Chicken, IB, Protectotype vaccination strategy, CMI, Humoral immunity

## Abstract

**Background:**

One of the most commonly applied protectotype vaccination protocol against infectious bronchitis (IB) in broiler chickens in the EU is simultaneous or alternate use of Ma5 and 4/91 vaccine strains. After IB vaccination and infection, systemic and upper respiratory tract (URT), humoral and cell-mediated immunity (CMI), are stimulated. The level of this stimulation correlates with the level of protection against IB.

**Results:**

We’ve investigated the development of URT and systemic, cell-mediated and humoral immunity in commercial broiler chickens vaccinated with Ma5 and/or 4/91 strains at hatch day. We’ve demonstrated that the group vaccinated with Ma5 and 4/91 strain simultaneously developed the most desirable immunity which reflects the level of CD8^+^ T cells stimulation in spleen and Harderian gland, as well as the level of IgA and IgY in URT washings and serum and their cross-reactivity with 7 IBV strains.

**Conclusions:**

Although we did not demonstrate directly why Ma5 + 4/91 protocol is so efficient it seems that it combines the benefits of monovalent vaccination with either Ma5 or 4/91 and while Ma5 seems to stimulate CMI more efficiently, the 4/91 strain generates a wider spectrum of immune system cross-reactivity and higher URT IgA production.

## Background

Infectious bronchitis (IB) is a highly contagious viral disease of chickens. IBV frequently changes its genotype and antigenic properties, tissue tropism, pathogenicity and eventually the course of the disease [1]. Due to the high level of antigenic variation among circulating viruses, establishing an effective vaccination schedule against IB has been difficult [1]. On the other hand, simultaneous or alternate use of Ma5 and 4/91, as commonly employed in the EU, induces high levels of protection against heterologous IBV types such as D1466 or QX. It has been demonstrated that this protocol doesn’t influence the development of protection against homologous IBVs while inducing high protection against heterologous IBV types such as D1466 or QX [2, 3].

After IB vaccination and infection, systemic and upper respiratory tract (URT), humoral and cell-mediated immunity (CMI), are stimulated. IBV are highly immunogenic and stimulate production of antibodies which can be detected with enzyme-linked immunosorbent assay (ELISA), serum neutralisation (SN) or haemaglutination inhibition (HI) test [2, 4, 5]. Although, humoral immunity can’t be considered a direct correlate of upper respiratory tract protection against IB, since the level of Igs in tears were not in correlation with the resistance to respiratory challenge, specific antibodies inhibit IBV replication in structures other than URT [1, 6–8].

Systemic CMI is considered as a decisive factor in protection against IB. Activation of cytotoxic cells correlated with lung and kidney IBV clearance [1]. Pei et al. [9] demonstrated that CD8^+^T cells isolated from spleens of birds, that recovered from IB and used for immunological transfer to naive chicks were capable of alleviating IB clinical course.

Little is known about the role of URT immunity in the protection against IBV. Several investigations indicated the role of innate immunity in the URT in the course of IB. Those mechanisms are not able to cure the infection, but they limit IBV replication and initiate specific immunity stimulation [10–12]. CD8^+^ T cells, capable of eliminating virus-infected cells, are mainly involved in the early stages of IB [5, 13] while CD4^+^ T cells and B lymphocytes are involved in later stages of specific immunity development [1, 4].

Considering the above, our own research was carried out in order to determine the development of URT and systemic, cell-mediated and humoral immunity following simultaneous immunization with the heterologous Ma5 and 4/91 IBV strains in commercial broiler chickens vaccinated at hatch day.

## Methods

The experimental procedures and animal handling procedures were conducted with the approval of the Local Ethics Committee for Animal Experiments in Olsztyn, Poland (approval number: 12/2015).

### Birds and vaccines

Experiments were conducted on 327 1-day old Ross 308 broiler chickens purchased from a commercial hatchery. Chickens were vaccinated in-ovo against Marek’s disease with bivalent (HVT + Rispens) vaccine. No additional vaccinations were performed at the hatchery. On day one blood samples and tracheal washings (TW) were collected from 23 randomly selected birds in order to establish the level of anti-IBV maternally derived antibodies (MDA). The rest of the birds were divided into 4 group (76 birds per group) as follows: control was the group that was not vaccinated against IB, Ma5 was the group vaccinated against IB at 1^st^ day of life (dol) with the use of Ma5 strain, 4/91 group was vaccinated at 1^st^ dol with 4/91 strain and V2 group was vaccinated at 1^st^ dol with both Ma5 and 4/91 strain in one diluent (sterile distilled water). Vaccination protocol was identical to the one described previously by Cook et al. [1] who (in one of the performed experiments) vaccinated SPF chickens with Ma5 and 4/91 strains at 1^st^ dol. All vaccines were prepared according to manufacturer instructions (Merck Animal Health, USA) and the birds were vaccinated oculo-nasally in a dose recommended by the manufacturer. Control group received sterile vaccine diluent at 1^st^ dol oculo-nasally.

Birds were raised till 21 dol in isolated units maintained at PCL 3 (physical containment level 3) facility. Water and feed were given to birds ad libitum.

### Experimental design

Birds were raised till 21 dol and during that period following samples were collected from birds of all of the experimental groups for laboratory evaluation. On 3, 7 and 14 days post vaccination (DPV) 5 samples of HGs (entire organ bilaterally; one sample was pooled from 3 birds, so 5 samples originated from 15 birds) and 5 spleen samples (individual) were collected for flow cytometry analysis of relative (in spleen; relative cell count—RCC) or absolute (in HG, absolute cell count—ACC) counts of CD4^+^ and CD8^+^ T as well as Bu1A^+^ B cells. Twenty three blood samples were collected at 14 and 21 DPV for serological evaluation of anti-IBV IgY in the serum. at 7, 14 and 21 DPV 15 samples of tracheal washings were collected for serological evaluation of specific anti-IBV IgY and IgA levels. Tracheal washings were collected by passing 1 ml of phosphate-buffered saline (PBS, pH 7.2, Sigma Aldrich, Germany) through the lumen of the trachea with a 2 ml syringe. The trachea was massaged gently to intensify the washing effect. TW and serum samples were stored at −20 °C until further analysis. Additionally, 5 serum samples reacting positive in ELISA at 21 DPV were subjected for HI analysis of cross-reactivity with different IBV diagnostic strains. Experimental design is summarized in Table 1.

**Table 1:** Experimental design—sampling time point

DPV	Blood (ELISA)	TW (ELISA)	HG (flow cytometry)	Spleen (flow cytometry)
3	-	-	+	+
7	-	+	+	+
14	+	+	+	+
21	+ (HI^a^)	+	-	-

On different DPV, samples were collected simultaneously from all of the experimental groups

^a^21 DPV 5 serum samples reacting possitive in ELISA were subjected for HI evaluation

### Flow cytometry

Isolation of mononuclear cells from HGs and spleens were performed as described previously [14]. HG and spleen samples (0,3 g +/− 10%) were homogenized in a manual Dounce tissue grinder and filtered (70 μm mesh). Cells pellets, obtained after centrifugation (450 g for 10 min at 20 °C), were resuspended in 3 ml of 40% Percoll density gradient and gently layered on 3 ml of 60% Percoll. Mononuclear cells were collected from the interphase after density centrifugation (20 min, 1900 g, 20 °C). Finally, the obtained mononuclear cells were resuspended in 1 ml of PBS. Vi-cell XR (Beckman Coulter, USA) cell counter and cell viability analyzer was used for each sample to calculate the absolute lymphocyte counts (ALC) per ml.

Half million (from HG) or 1 × 10^6^ (from spleens) of viable mononuclear cells were stained (3 μl/1 × 10^6^ cells) with monoclonal mouse anti-Chicken CD4 FITC (0,1 mg/ml) and mouse anti-chicken CD8 RPE (0,1 mg/ml) or mouse anti-chicken Bu1a FITC (0,1 mg/ml) antibodies (AbD Serotec, UK), incubated for 30 min on ice, washed twice in PBS and analyzed with the FACSCanto II (BD, USA) flow cytometer.

Further steps of flow cytometry data acquisition and dual platform analysis allowing the calculation of CD4^+^ and CD8^+^ T and Bu1a^+^ B RCC in the spleen and CD4^+^, CD8^+^ T and Bu1a^+^ B ACC in HG samples were performed as described previously [14]. ACC of T and B cells for HG samples were calculated with the use of the following formula: ACC = (ALC * RCC)/100%. Data were expressed as mean RCC (for spleen samples) or ACC (for HG samples) of different T or B cell subpopulations +/− SD.

### ELISA

ELISA evaluation was performed with the use of commercial IBV ELISA kit (IDEXX, USA) according to manufacturer recommendation with minor modifications. Serum samples collected from birds 14 and 21 DPV were diluted 1:100 (instead of 1:500) in order to increase low antibody level detection. TW were tested undiluted and the level of specific IgA in TW was detected as described previously [15] with minor modifications. Anti-IBV IgA antibodies were detected by replacing the conjugated antibodies of ELISA kit with goat anti-chicken IgA HRP antibodies (AbD Serotec, UK) dilluted 1:10 000. The following steps of ELISA procedure were performed with Eppendorf epMotion 5075 LH automated pipetting station (Eppendorf, Germany), BioTek ELx405 automatic plate washer (BioTek, USA) and BioTek ELx800 plate reader. Based on sample ODs the sample to positive (S/P)-ratios were calculated and used to express the mean serum and TW anti-IBV antibody (S/P)-ratio +/− SD per group and day post vaccination. Mean OD values +/− SD per group and day post vaccination were used to express the level of anti-IBV IgA antibodies in TW.

### HI test

HI test was performed with M41, 793B, D3128, Italy O2, D8880, D274, D1466 HA antigens obtained from GD Animal Health Service (Deventer, Netherlands). The HI test was conducted according to standard procedure (OIE) using 4 HA units of antigen per well. The result of HI analyzes are express as mean HI antibody titer (log_2_ base) +/− SD.

### Statistical analysis

Statistical analysis of flow cytometry results was performed with GraphPad Prism 6.05 with the use of student T - test. Differences were considered statistically significant if *p* < 0,05 and highly significant if *p* < 0,01. Statistical analysis of serological evaluation (ELISA, HI) results was performed with Statistica Pl v.10 with the use of nonparametric Kruskal-Wallis test. Differences were considered statistically significant if *p* < 0,05.

## Results and discussion

At the beginning of the experiment chicks had high anti-IBV MDA levels both in serum (mean S/P ratio = 2.349 +/− 1.17) and TW (mean OD = 0.203 +/− 0.452) and the level of these antibodies declined gradually in the control group (Fig. 3a and b). MDA have been demonstrated to interfere with immune response after IB vaccination. For example, Terregino et al. [3] demonstrated that commercial chickens vaccinated against IB with the use of Ma5 and 4/91 strains (with 14 days interval between vaccinations) developed lower titers of HI antibodies in comparison with SPF birds. Nevertheless, in this trial, both SPF and commercial chickens had significantly increased HI antibody titers in comparison with the adequate control groups. Additionally, Chhabra et al. [16] demonstrated that MDA do not influence the production of IgA in URT after vaccination (with either H120 strain alone or in combination with CR88) of day old commercial broiler chickens. In this experiment infiltration of different subpopulations of T and B cells in the URT was also unaltered and consistent with SPF birds. Overall, it seems that maternal immunity, to some extent, may neutralize the vaccine virus (especially when given to birds at the hatch day) but the general outcome of immune system stimulation after IB vaccination is unaltered by the MDA.

Both Ma5 and 4/91 vaccine strains belong to a different, antigenically distinct serotypes. Ma5 is based on the Massachusetts (Mass) serotype and the 4/91 strain belongs to 4/91 serotype. Wild-types of those viruses are known for their different tissue tropism, pathogenicity and most importantly antigenic structure. IBV serotyping is done based on the S1 protein amino acid sequence analysis. The basic hypothesis of protectotype vaccination strategy, presented by Cook et al. [2], was that - new IBV serotypes can arise as a result of only a very few changes in the amino acid composition of the S1 part of IBV spike protein. On the other hand remaining part of the virus antigen uniform remains unchanged. Those shared antigens can induce cross protective immunity against heterologous viruses. As it turned out, the combination of Ma5 and 4/91 vaccine strains for vaccination of chickens resulted in greater cross protection against heterologous IB viruses in comparison to monovalent vaccination with either Ma5 or 4/91 strain [2]. However, up to date, the underlying immune mechanism that could explain the efficacy of this protocol is unknown.

### Cellular immune response

Considering the fact that CMI associated mainly with cytotoxic T cells is protective against IBV we have performed flow cytometry analysis of both URT and systemic cellular immune response after different IB vaccination protocols.

#### Ma5 group

**Fig. 1:**
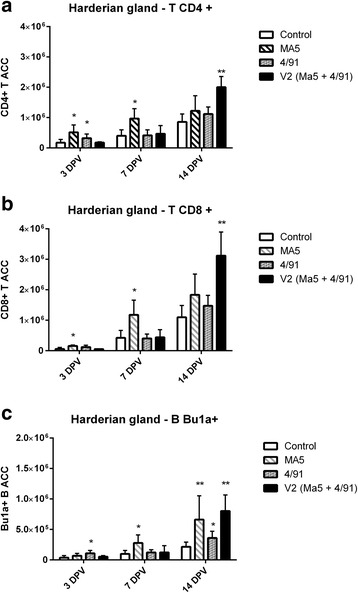
Mean CD4^+^ (**a**) and CD8^+^ (**b**) T and Bu1a^+^ B (**c**) ACC +/− SD in HG samples in different groups and day post vaccination. ^*^ Significant differences at different DPV (T-test, ^*^ as *p* < 0,05) in comparison to the control group. ^**^ Significant differences at different DPV (T-test, ^**^ as *p* < 0,01) in comparison to the control group

In the Ma5 group statistically significant increase of CD4^+^ and CD8^+^ T ACC in HG was recorded 3 and 7 DPV (Fig. 1a and b). Additionally, in the Ma5 group significant or highly significant increase of Bu1A^+^ B ACC in HG was recorded 7 and 14 DPV (Fig. 1c) respectively in comparison to the control group.

**Fig. 2:**
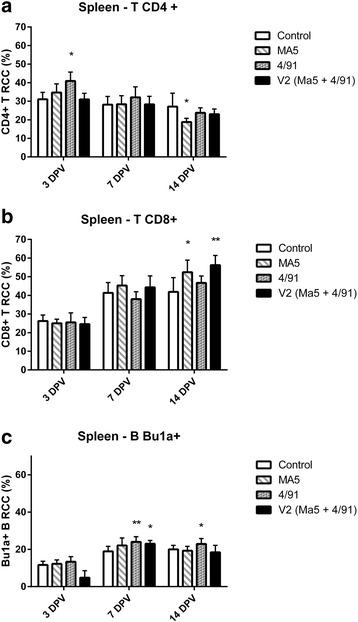
Mean CD4^+^ (**a**) and CD8^+^ (**b**) T and Bu1a^+^ B (**c**) RCC +/− SD in spleen samples in different groups and day post vaccination. ^*^ Significant differences at different DPV (T-test, ^*^ as *p* < 0,05) in comparison to the control group. ^**^ Significant differences at different DPV (T-test, ^**^ as *p* < 0,01) in comparison to the control group

Statistically significant decrease of CD4^+^ T RCC was recorded in the spleen 14 DPV (Fig. 2a) in the Ma5 group. Additionally in this group, significant increase of CD8^+^ T RCC (Fig. 2b) in the spleen was recorded 14 DPV in comparison to the control group.

#### 4/91 group

In 4/91 group statistically significant increase of CD4^+^ T ACC was recorded 3 DPV in HG (Fig. 1a). In this group we’ve recorded a significant increase of Bu1A^+^ B ACC in HG 3 and 14 DPV (Fig. 1c) in comparison to the control group.

Significant increase of CD4^+^ T RCC in the spleen was recorded 3 DPV in 4/91 group (Fig. 2a). In this group we’ve recorded a significant and highly significant increase of Bu1A^+^ B RCC, 7 and 14 DPV (Fig. 2c), in comparison to the control group.

#### V2 group

In V2 group a highly significant increase of both CD4^+^ T and CD8^+^ T ACC as well as Bu1A^+^ B ACC in HG was recorded 14 DPV (Fig. 1a, b and c).

In V2 group a highly significant increase of CD8^+^ T RCC in the spleen was recorded 14 DPV (Fig. 2b). Additionally, in this group a significant increase of Bu1A^+^ B RCC was recorded 7 DPV in spleen (Fig. 2c).

From presented data it turns out that the fastest stimulation of CMI parameters (associated with cells harboring CD8 molecule) in HG was recorded in Ma5 group. Despite the delayed character of this stimulation the highest infiltration of immunocompetent cells in URT was recorded 14 DPV in V2 group. Unfortunately, we did not perform further analyzes to determine the actual level of lymphocytes stimulation but if we consider time dependencies of CD8 marker expression we may distinguish early and late CMI stimulation. And while early, probably associated mainly with the activation of NK cells, was most prominently stimulated in Ma5 group, the late stimulation connected with cytotoxic lymphocytes activity was strongest in V2 group.

The results of our study corroborates previous findings of Chhabra et al. [16] who demonstrated that CD4^+^ and CD8^+^ cells infiltration in the URT takes place in first 2 weeks after IB vaccination of day old broiler chickens and that the overall number of infiltrating CD8^+^ cells is greater in comparison to the number CD4^+^ cells. Additionally, cited authors demonstrated that broilers with higher number of infiltrating CD8^+^ cells in the URT (on the day of IB infection) developed greater protection against IBV challenge.

Considering time dependencies between CD4^+^ T cells and B cells contribution in HG we may speculate that mainly Th2 subpopulation (involved in humoral immunity stimulation through cytokines production) of T helper cells was stimulated after IB vaccination.

Similar time dependencies between CD4^+^ T cells and B cells was also recorded in the spleens of vaccinated birds. The strongest stimulation of those parameters was recorded in 4/91 group but also V2 which may have influenced the results of HI test which will be discussed later.

The contribution of T CD8^+^ cells in the spleen increased in all of the IB vaccinated groups (significant increase was recorded in Ma5 and V2 group), but the strongest stimulation of this parameter was recorded in V2 group. Pei et al. [9] demonstrated that CD8^+^ T cells isolated from spleens of birds, that recovered from IB and used for immunological transfer to naive chicks were capable of alleviating IB clinical course.

### Humoral immune response

**Fig. 3:**
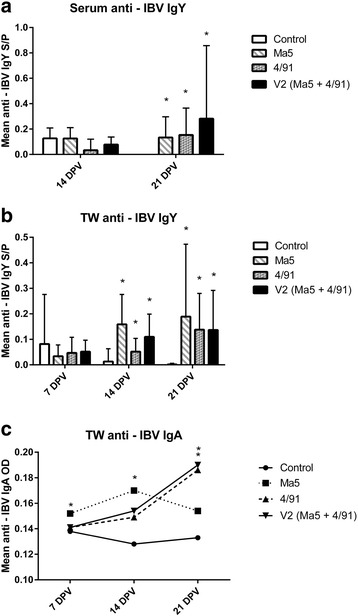
Mean IgY serum level (**a** - data expressed as mean S/P), IgY TW level (**b** - data expressed as mean S/P) and IgA TW level (**c** - data expressed as mean OD) in different groups and day post vaccination. ^*^ Significant differences at different DPV (Kruskal-Wallis test, ^*^ as *p* < 0,05) in comparison to the control group

A statistically significant increase of serum IgY level was recorded in all of the vaccinated group (in comparison to the control group) 21 DPV while the highest mean level of anti-IBV IgY was recorded, at this time, in V2 group (Fig. 3a).

Similarly, a statistically significant increase of mean TW IgY level was recorded in all of the vaccinated group (in comparison to the control group) 14 and 21 DPV, while the highest mean level of anti-IBV IgY 21 DPV was recorded in Ma5 group (Fig. 3b).

In Ma5 group a significant increase of specific IgA mean levels was recorded 7 and 14 DPV (in comparison to the control group). In comparison, in 4/91 and V2 group an increase anti-IBV IgA in TW was recorded 14 and 21 DPV while the highest mean level of anti-IBV IgA 21 DPV was recorded in V2 group (Fig. 3c).

Okino et al. [4] demonstrated a gradual increase of specific IgA and IgG in the URT washings with simultaneous activation of CMI parameters in the URT after single full-dose vaccination of newly hatched chicks with H-120 IBV strain. In addition, these humoral and CMI response evaluated at mucosal sites correlated significantly with tracheal protection against homologous IBV challenge. Additionally, our findings demonstrate that there are different patterns of IgA levels in tracheal washes after different IB vaccination protocol application, which indicates that 4/91 strain alone or in combination with Ma5 strain (V2 group) generates longer lasting and higher IgA levels in TW.

HI test results are summarized in Table 2. In Ma5 group no statistical differences was recorded in mean HI antibody titer against any of the homologous or heterologous IBV strains in comparison to the control group. In 4/91 group a statistically significant increase of mean HI antibody titer against 5 out of 7 IBV strains (without It-O2 and D8880) was recorded while in two cases (against 4/91 and D3128 strains) it was also significantly higher in comparison to Ma5 group. In V2 group a statistically significant increase of mean HI antibody titer against D8880 IBV strain was recorded in comparison to the control group.

**Table 2:** Infectious bronchitis HI mean antibody titre (log_2_ base) +/− SD, 21 DPV

Group		IBV diagnostic strain						
		M41	4/91	D3128	Italy O2	D8880	D274	D1466
Control	HI titer	2,4^b^	3,0^b^	2,0^b^	2,25^a^	3,0^b^	2,0^b^	2,8 ^b^
	SD	0,55	0,00	0,00	0,50	0,71	0,00	0,84
Ma5	HI titer	3,2^a, b^	3,22^b^	2,0 ^b^	3,33 ^a^	3,6 ^a, b^	3,0 ^a, b^	4,6 ^a, b^
	SD	0,45	0,45	0,00	0,58	0, 55	0,71	1,34
4/91	HI titer	4,25 ^a^	5,42^a^	3,5 ^a^	3,5 ^a^	4,0 ^a, b^	5,0^a^	6,8^a^
	SD	0,96	0,55	0,71	0,71	0,71	1,41	1,64
V2	HI titer	3,8^a, b^	4,2^a, b^	2,67^a, b^	2,6 ^a^	4,8^a^	3,5 ^a, b^	4,5 ^a, b^
	SD	0,45	0,00	0,58	0,55	0,45	0,58	1,30

^a,^ ^b^—Mean values in a column with different superscript letters are significantly different (Kruskal-Wallis test, *p* < 0,05)

Overall percentage of samples reacting positive (neg/pos cut-off > 3 log_2_ HI titer) with different IBV strains was 38.71, 77.42 and 59.38% in Ma5, 4/91 and V2 group, respectively.

## Conclusions

It has been demonstrated previously that Ma5 + 4/91 IB vaccination protocol doesn’t influence the development of protection against homologous IBV strains (Ma5 or 4/91) while inducing high protection against heterologous IBV types [2, 3]. Although 14 days interval between Ma5 (1 dol) and 4/91 (14 dol) vaccination protocol results in even better protection (especially against heterologous IBV types) than Ma5 and 4/91 vaccines given to birds simultaneously at 1 dol [2], we’ve decided to use this protocol in order to: (1) uniform birds’ age within the experimental groups and to (2) imitate the widespread use of this protocol under field conditions. It has been demonstrated that both vaccination protocols (Ma5 + 4/91 at 1st dol or Ma5 at 1st dol and 4/91 at 14th dol) generates comparable protection against experimental infection with either homo- or heterologous IBV serotype and in both cases the level of protection was greater and wider against heterologous IBV strains in comparison to Ma5 or 4/91 alone vaccination protocol [2].

The aim of our study was to evaluate the immune mechanisms that could explain the efficiency of Ma5 + 4/91 vaccination protocol. From our research it seems that this protocol combines the benefits of monovalent vaccination with either Ma5 or 4/91. Both of those strains are immunogenic, but while Ma5 alone seems to stimulate CMI (especially in the URT) more efficiently the 4/91 strain generates a wider spectrum of immune system cross-reactivity and higher URT IgA production. Eventually, the final answer could be that this efficiency results from additive impact of Ma5 and 4/91 strains on different levels of both innate and acquired host immune response.

## Abbreviations

ACC: Absolute cell count
CMI: Cell mediated immunity
DOL: Day of life
DPV: Day post vaccination
ELISA: Enzyme-linked immunosorbent assay
HA: Haemagglutination
HG: Harderian gland
HI: Haemaglutination inhibition
IB: Infectious Bronchitis
IBV: Infectious Bronchitis virus
MDA: Maternally derived antibodies
PCL: Physical containment level
RCC: Relative cell count
SN: Serum neutralisation
TW: Tracheal washings
URT: Upper respiratory tract
